# Medicinal Plants in the Prevention and Treatment of Chronic Diseases 2013

**DOI:** 10.1155/2014/180981

**Published:** 2014-03-26

**Authors:** Mohamed Eddouks, Debprasad Chattopadhyay, Vincenzo De Feo, William Chi-shing Cho

**Affiliations:** ^1^Moulay Ismail University, FST Errachidia, BP 21, 52000 Errachidia, Morocco; ^2^ICMR Virus Unit, Division of Ethnomedicine, ID & BG Hospital, General Block 4, 57 Dr. Suresh C. Banerjee Road, Kolkata 700 010, India; ^3^Dipartimento di Farmacia, Università degli Studi di Salerno, Via Giovanni Paolo II 132, Fisciano, 84084 Salerno, Italy; ^4^Department of Clinical Oncology, Queen Elizabeth Hospital, Kowloon, Hong Kong

Since time immemorial, in search for rescue for their disease, people looked for drugs in nature. The traditional use of medicinal plants can lead to the discovery of new potent botanical agents in the treatment of several diseases. Some 7000 natural compounds are currently used in modern medicine; most of these had been used for centuries by traditional healers and the global market value of medicinal plant products exceeds $100 billion per annum. In spite of the development of pharmacological agents for the treatment of chronic diseases, the use of medicinal plants continues to flourish. Over the last century, the drastic changes of human life style and eating habits lead to the emergence of various chronic diseases. The decreasing efficacy of some synthetic drugs and the increasing contraindications of their usage make the usage of natural drugs topical again. Thus, the study of phytotherapy for chronic diseases treatment might yield an excellent return in potential sources of medicinal plants which play vital roles in disease prevention and their promotion and use fit into all existing prevention strategies. In this issue, we aim to present some recent advances in the use of medicinal plants for treating the chronic diseases such as diabetes, cancer, cardiovascular diseases, inflammation, and neurologic disorders.

In a clinical study, “Effect of eucalyptus oil inhalation on pain and inflammatory responses after total knee replacement: a randomized clinical trial,” Y. S. Jun et al. demonstrate the beneficial effects of eucalyptus oil inhalation on pain and inflammatory responses after total knee replacement surgery. S. Zhang et al., “Skimmin, a coumarin from* Hydrangea paniculata*, slows down the progression of membranous glomerulonephritis by anti-inflammatory effects and inhibiting immune complex deposition,” investigated the renoprotective activity of skimmin, one of the major pharmacologically active molecules present in* Hydrangea paniculata*. They studied also the underlying mechanisms of the observed renoprotective effects of skimmin in a rat model of membranous glomerulonephritis induced by cationic bovine serum albumin which may be the inhibition of IL1*β* and IL-6 expression. In another study “Traditional Chinese medicine Tang-Luo-Ning ameliorates sciatic nerve injuries in streptozotocin-induced diabetic rats,” D.-W. Zou et al. describe the beneficial effect of Tang-Luo-Ning (TLN), an effective traditional Chinese medicine for the treatment of diabetic peripheral neuropathy (DPN). To illustrate the underlying neural protection mechanisms of TLN, the effect of TLN on electrophysiology and sciatic nerve morphology was investigated in a model of streptozotocin-induced DPN. G. Belcaro et al., “Grape seed procyanidins in pre- and mild hypertension: a registry study,” studied the efficacy of a standardized grape seed procyanidins extract in decreasing blood pressure when associated with nondrug intervention (diet and lifestyle modification) in a controlled registry study involving healthy prehypertensive and mildly hypertensive subjects. The authors supported that the effect on blood pressure adds to the beneficial effects of grape seed procyanidins on the cardiovascular disease phenotype associated with the oxidation of membrane lipids (endothelial disfunction, formation of oxidized LDL, and activation of phagocytic cells). A study titled (“Effect of* Nelumbo nucifera* petal extracts on lipase, adipogenesis, adipolysis, and central receptors of obesity”) by C. C. Velusami et al. demonstrates that both methanol and successive water extracts of* Nelumbo nucifera* petals had an effect on inhibition of lipid storage in adipocytes and increasing lipolysis. In addition, methanol extract exhibited the concentration dependent inhibitory effect on lipase activity. Furthermore,* Nelumbo nucifera* petal extracts showed evident agonist and antagonist activity towards serotonin and cannabinoid receptors, respectively, while it showed no effects towards melanocyte concentrating hormone and melanocortin receptors. Another clinical study performed by G. Belcaro et al. “Greenselect Phytosome for borderline metabolic syndrome” demonstrates that Greenselect Phytosome, a proprietary lecithin formulation of a caffeine-free green tea catechin extract, was especially effective for weight/waist changes in a controlled registry study on asymptomatic subjects borderline for metabolic syndrome factors and with increased plasma oxidative stress. A. Hunyadi et al., “Metabolic effects of mulberry leaves: exploring potential benefits in type 2 diabetes and hyperuricemia,” report a series of relevant* in vitro* and* in vivo* studies on the bioactivity of an extract of mulberry leaves and its fractions.* In vivo* antihyperglycemic and antihyperuricemic activity, plasma antioxidant status,* in vitro* glucose consumption by adipocytes in the presence or absence of insulin, xanthine oxidase inhibition, free radical scavenging activity, and inhibition of lipid peroxidation were analyzed.

In addition, known bioactive constituents of mulberry were identified and quantified. A study performed by H.-M. Zhao et al., “Si Shen Wan inhibits mRNA expression of apoptosis-related molecules in p38 MAPK signal pathway in mice with colitis,” demonstrated that Si Shen Wan, a formula of traditional Chinese medicine used to treat ulcerative colitis, allergic colitis, and chronic colitis, effectively inhibited mRNA expression of apoptosis-related molecules in p38 MAPK signal pathway to downregulate colonic epithelial cells apoptosis in colonic mucosa from mice with colitis. In another study “Therapeutic potential of andrographolide isolated from the leaves of* Andrographis paniculata* nees for treating lung adenocarcinomas,” Y.-T. Tung et al. have studied the antipulmonary cancer effects of andrographolide in a lung tumor mouse model induced by human vascular endothelial growth factor A165 (hVEGF-A165). The antiangiogenesis and chemotherapeutic potential of andrographolide may provide a cure for pulmonary tumors in the future. Z.-Z. Meng et al., “Effect of Xiaoyaosan decoction on learning and memory deficit in rats induced by chronic immobilization stress,” observed the effect of Xiaoyaosan (XYS) decoction on chronic immobilization stress- (CIS-) induced learning and memory deficit in rats from behaviors and changes of proteins in hippocampus. The findings suggested that XYS decoction may be helpful in reversing CIS-induced learning and memory deficit by increasing the levels of postsynaptic density protein 95 and synaptophysin on the hippocampal nerve synapses and improving synaptic plasticity.

A review conducted by L. O. Chuah et al., “Updates on antiobesity effect of* Garcinia* origin (–)-HCA,” summarizes the update of chemical constituents, significance of* in vivo*/clinical antiobesity effects, and the importance of the current market potential of* Garcinia*/hydroxycitric acid especially as a potential supplement for weight management and as antiobesity agent. A study realized by Y. Liang et al., “Effects of the Chinese traditional prescription Xiaoyaosan decoction on chronic immobilization stress-induced changes in behavior and ultrastructure in rat hippocampus,” demonstrated the potential mechanism of Xiaoyaosan (XYS) decoction's antidepressant-like effect in *α*-amino-3-hydroxy-5-methyl-4-isoxazolepropionic acid (AMPA) receptors related to synaptic plasticity in the hippocampus rats induced by chronic immobilization stress. The study demonstrates that XYS decoction may produce an antidepressant-like effect, which appears to be involved with AMPA receptors related synaptic plasticity of hippocampus. In another study “*Citrus Bergamia* Risso elevates intracellular Ca^2+^ intracellular stores in human umbilical vein endothelial cells,” G. H. Seol et al. demonstrate that Citrus Bergamia Risso mobilizes Ca^2+^ from primary intracellular stores via Ca^2+^-induced and IP3-mediated Ca^2+^ release and affect promotion of Ca^2+^ influx, likely via an store-operated Ca^2+^ mechanism. This finding supports the potential roles of bergamottin in cardiovascular function. J. Hoscheid et al., “Inhibitory effect of the hexane fraction of the ethanolic extract of the fruits of* Pterodon pubescens* Benth in acute and chronic inflammation,” confirm the anti-inflammatory activity of the hexane fraction of an ethanolic extract of* Pterodon pubescens* Benth. The anti-inflammatory activity was measured with increasing doses of the hexane fraction by using a carrageenan-induced rat model of pleurisy and a rat model of complete Freund's adjuvant-induced arthritis. The results of biochemical, hematological, and histological analyses indicated a significant decrease in glucose, cholesterol, and triglycerides levels and reduction in the number of total leukocytes and mononuclear cells. The study demonstrates also the absence of toxicity for the doses used. H. Park et al., “Ampelopsis Radix protects dopaminergic neurons against 1-methyl-4-phenylpyridinium/1-methyl-4-phenyl-1,2,3,6-tetrahydropyridine-induced toxicity in Parkinson's disease models* in vitro* and* in vivo*,” have demonstrated that Ampelopsis Radix has neuroprotective effects with antioxidant mechanisms in Parkinson's disease models. The standardized extract of Ampelopsis Radix protected dopaminergic neurons by inhibiting reactive oxygen species generation* in vitro*, showed potent radical scavenging activities* in vitro*, and protected the dopaminergic neurons in the brain in the mouse Parkinson's disease model leading to motor improvements. Another paper by H. Ha et al., “Antiatopic dermatitis effect of* Artemisia iwayomogi* in dust mice extract-sensitized Nc/Nga mice,” demonstrates the anti-inflammatory and antiatopic dermatitis effects of* Artemisia iwayomogi* both* in vitro* and* in vivo*.* Artemisia iwayomogi* inhibited the nitric oxide and histamine productions in RAW264.7 and MC/9 cells. Furthermore, isochlorogenic acid A, chlorogenic acid, and scopoletin were demonstrated to be the major components of this plant. Additionally, in the mice, the topical application of* Artemisia iwayomogi* reduced the dermatitis scores in the dorsal skin and ears and reduced the plasma levels of IgE. E. Panzarini et al., “Administration dependent antioxidant effect of* Carica papaya* seeds water extract,” demonstrate that* Carica papaya* seeds water extract is potentially useful for protection against oxidative stress. The authors have assessed the antioxidant activities of the* Carica papaya* seeds water extract against hydrogen peroxide oxidative stress in human skin Detroit 550 fibroblasts. An* in vivo* study by M.-H. Chen et al., “Antidiabetic and antihyperlipidemic effects of* Clitocybe nuda* on glucose transporter 4 and AMP-activated protein kinase phosphorylation in high-fat-fed mice,” demonstrates that amelioration of diabetic and dyslipidemic state by* Clitocybe nuda* extract in high-fat-fed mice occurred by regulation of GLUT4, glucose-6-phosphatase, and AMP-activated protein kinase phosphorylation. The plant extract decreased hepatic glucose production in the liver and enhanced glucose uptake in skeletal muscle. This study presents a deep analysis of mechanisms of action involved in the antidiabetic and antihyperlipidemic activities of* Clitocybe nuda*.

After the first volume of this special issue published in 2012, we hope that this issue will present valuable information for scientists and clinicians.

